# Glucose variability measured by continuous glucose monitoring is associated with skin autofluorescence: the Maastricht Study

**DOI:** 10.1007/s00125-025-06469-5

**Published:** 2025-06-20

**Authors:** Nefeli M. Dimitropoulou, Simone J. P. M. Eussen, Casper G. Schalkwijk, Bastiaan E. de Galan

**Affiliations:** 1 Department of Internal Medicine, Maastricht University Medical Centre+, Maastricht, the Netherlands; 2 Cardiovascular Research Institute Maastricht (CARIM), Maastricht University, Maastricht, the Netherlands; 3 Department of Epidemiology, Maastricht University, Maastricht, the Netherlands; 4 Care and Public Health Research Institute (CAPHRI), Maastricht University, Maastricht, the Netherlands; 5 Department of Internal Medicine, Radboud University Medical Centre, Nijmegen, the Netherlands

**Keywords:** AGE reader, Cardiovascular disease, Coefficient of variation metric, Diabetes, Skin advanced glycation end-products, Standard deviation metric

## Abstract

**Aims/hypothesis:**

Glucose variability in people with type 2 diabetes has been associated with increased risk of CVD, and AGEs might be an underlying mechanism. Therefore, this study investigates associations of glucose variability with AGEs in the skin in people with and without impaired fasting glucose, impaired glucose tolerance or diabetes.

**Methods:**

We used data from the Maastricht Study, a population-based cohort study. Glucose variability and AGEs in skin were measured by continuous glucose monitoring (CGM) and skin autofluorescence (SAF), respectively. Multiple linear regression was used to test the association of CGM-metrics CV and SD with SAF and adjusted for age, sex, CVD risk factors, nutritional factors and educational level. Interaction analysis was used to test the effect of glucose metabolism status on the association of CV and SD with SAF.

**Results:**

We included 795 participants (mean ± SD age 59 ± 8.7 years; 49% were female). Glucose metabolism status was stratified into normal glucose metabolism (*n *= 459), prediabetes (*n *= 174) and type 2 diabetes (*n *= 162). Individuals with type 2 diabetes had higher values of SAF (mean ± SD 2.3 ± 0.6 arbitrary units [AU]) than those with prediabetes (2.1 ± 0.4 AU, *p *= 0.014) and normal glucose metabolism (2.0 ± 0.4 AU, *p *= 0.007). In the cohort, both SD (0.152 AU [IQR 0.088–0.217]) and CV (0.014 AU [IQR 0.005–0.017]) were significantly associated with SAF in fully adjusted analyses. Glucose metabolism status did not modify the associations of SD and CV with SAF.

**Conclusions/interpretation:**

A higher glucose variability is associated with higher levels of SAF, suggesting that glucose variability plays a role in the formation of AGEs.

**Graphical Abstract:**

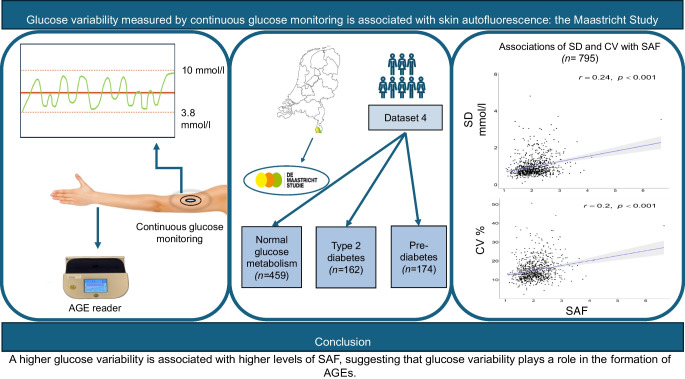

**Supplementary Information:**

The online version contains peer-reviewed but unedited supplementary material available at 10.1007/s00125-025-06469-5.


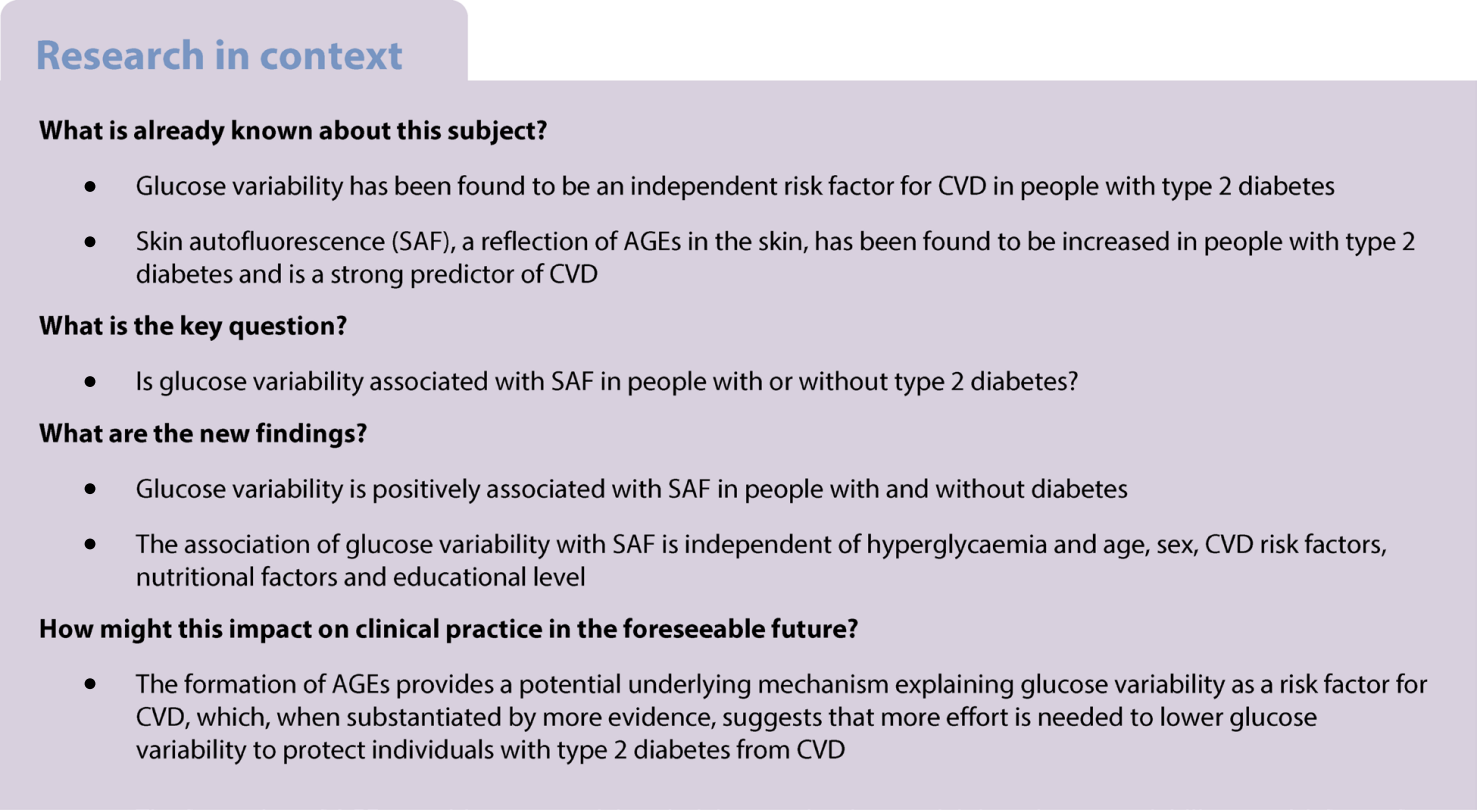


## Introduction

Globally, the incidence of type 2 diabetes increases due to ageing of the population and the obesogenic environment [1]. Type 2 diabetes carries a high risk of multiple complications, with CVD being the predominant cause of disability and death among people with diabetes [1, 2]. Both chronic and postprandial hyperglycaemia are independent risk factors for the occurrence of both microvascular complications and CVD in diabetes [3, 4]. In fact, even mildly elevated glucose, as can be found in prediabetes (i.e. impaired fasting glucose or impaired glucose tolerance), has been associated with increased risks of cardiovascular morbidity and mortality [5].

More recently, glucose variability, as measured by continuous glucose monitoring (CGM), has been associated with CVD risk [6]. Moreover, a recent meta-analysis suggested that elevated glucose variability is also associated with the development of atherosclerosis in people without diabetes [7]. Glucose variability, typically displayed as CV over 2 weeks in standard CGM downloads, is defined as the sum of (postprandial) glycaemic excursions and (minor) hypoglycaemia can reflect both within-day and across-day variability [7]. As a metric, it provides additive information on top of HbA_1c_, which despite being the gold standard for overall glycaemic control, does not reflect the variation in glycaemic profile [8].

The underlying mechanism(s) explaining how glucose variability translates into elevated risks of CVD in people with diabetes is not entirely clear but formation of AGEs may play an important role. Dicarbonyls including methylglyoxal are major precursors of AGEs and are directly derived from reactive glucose metabolites. AGEs are elevated in people with diabetes and can lead to tissue damage and subsequent complications [9]. There is a clear link between hyperglycaemia and AGEs but whether and to what extent AGEs are elevated in relation to high glucose variability has not been investigated.

Skin autofluorescence (SAF) is an indirect non-invasive method for measuring AGEs in the skin. Elevated SAF is a strong predictor of CVD [10]. SAF has been found to be increased in people with type 2 diabetes [10] and may reflect the formation of AGEs directly from glucose in the interstitium. The latter is of particular interest since CGM measures glucose in the interstitial fluid. Therefore, measuring SAF in people wearing a glucose sensor provides a unique opportunity to gain a better insight into the role of elevated glucose and glucose variability in the formation of AGEs, both measured in the same compartment [11].

We therefore investigated the association of glucose fluctuations, when measured in the subcutaneous tissue by CGM, with SAF in people with and without type 2 diabetes.

## Methods

### Study population

We used data from the Maastricht Study, an observational prospective population-based cohort study. The rationale and methodology have been described previously [12]. In brief, the study focuses on the aetiology, pathophysiology, complications and comorbidities of type 2 diabetes and is characterised by an extensive phenotyping approach. Eligible for participation were all individuals aged between 40 and 75 years and living in the southern part of the Netherlands. Participants were recruited through mass media campaigns and from the municipal registries and the regional Diabetes Patient Registry via mailings. Recruitment was stratified according to known type 2 diabetes status, with an oversampling of individuals with type 2 diabetes, for reasons of efficiency. The examinations of each participant were performed within a time window of 3 months. The Maastricht Study has enrolled 9187 participants in total between November 2010 and October 2020. The current analysis includes cross-sectional data from 900 participants. Participants with missing data on CGM, SAF measurements and other types of diabetes (e.g. type 1 diabetes) were excluded.

The Maastricht Study was approved by the institutional medical ethical committee (NL31329.068.10) and the Minister of Health, Welfare, and Sports of the Netherlands (Permit 131088-105234-PG). All participants gave written informed consent [12].

### Assessment of diabetes status

Participants underwent a standardised 2 h 75 g OGTT after being fasted overnight. Participants with a fasting plasma glucose level above 11 mmol/l, determined by a finger prick, were not allowed to undergo the OGTT for safety reasons. The diagnosis of those participants was based on the use of glucose-lowering medication and the fasting plasma glucose. Based on the WHO 2006 criteria, glucose metabolism status was defined as normal glucose metabolism (fasting plasma glucose <6.1 mmol/l), prediabetes (fasting plasma glucose ≥6.1 mmol/l and ≤7.0 mmol/l) and type 2 diabetes (fasting plasma glucose ≥7.0 mmol/l) [13].

### Assessment of glucose variability

Participants willing to do so, were asked to wear a CGM device (iPro2 and Enlite Glucose Sensor; Medtronic, Tolochenaz, Switzerland) for 1 week (*n *= 869) [14]. For the assessment of glucose variability, we used the SD and the CV. We also collected data on time in range (TIR), time above range (TAR, >10 mmol/l) and time above tight range (TATR), which was defined as glucose values above 7.8 mmol/l. This latter metric was preferred over the more conventional TAR, because of the paucity of the latter, particularly in participants without diabetes [15]. Finally, we also included the mean amplitude of glycaemic excursions (MAGE), defined as an index of glycaemic variability [16], and mean of daily differences (MODD), which is the mean of all valid absolute value differences between glucose concentrations that were measured at the same time of day on two consecutive days [17].

### Skin autofluorescence

The AGE reader (DiagnOptics Technologies) was used to measure AGEs in the skin. This is a desktop device that estimates the level of AGE accumulation in the skin by using the characteristic fluorescent properties of certain AGEs. Technical information of this non-invasive method has been described previously [18]. In brief, the AGE reader device illuminates a skin surface of 4 cm^2^ guarded against surrounding light, with an excitation λ range of 300–420 nm and with a peak excitation λ of 370 nm. The calculation of SAF is based on the ratio between the emission light from the skin in the λ range of 420–600 nm (fluorescence) and excitation light that is reflected by the skin (λ range 300–420 nm), multiplied by 100 and expressed in AU. Offline calculations of SAF were done by automated analysis using AGE Reader software, version 2.3, and was observer independent [19].

### Measurements of covariates

Information regarding age, biological sex, educational level (low, medium, high), history of CVD and smoking status (never, former, current smoker) was obtained through questionnaires. Medication use was assessed via interviews that are described elsewhere [12]. Physical examination was performed to measure body weight, height, waist circumference and systolic and diastolic BP. Blood samples were obtained to measure fasting glucose, HbA_1c_ and lipid profile. Dietary intake was assessed using the food frequency questionnaire (FFQ) as described elsewhere [12]. The Dutch Food Composition Database is a tool that estimates the intake of total energy and individual mono- and disaccharides [13]. The Dutch Health Diet (DHD) includes the 15 food-based Dutch dietary guidelines. The DHD was updated in 2015 and consists of 15 items [20] and because the FFQ does not contain information on filtered or unfiltered coffee, the DHD includes 14 components (with a maximum of 140 points).

### Statistical analysis

Normally distributed continuous data are presented as mean ± SD, the non-normally distributed data as median (IQR) and categorical data as *n* (%). Data are presented in the total study population, as well as stratified by glucose metabolism group (i.e. type 2 diabetes, prediabetes or normal glucose metabolism). ANOVA was used for the comparisons between the three glucose metabolism groups and Tukey test for pairwise comparisons. The Mann–Whitney *U* test was used for the non-normally distributed data (CGM data, total cholesterol:HDL-cholesterol ratio, HbA_1c_). The χ^2^ test was used for categorical variables. For exploratory purposes, we investigated the correlation of glucose variability using CV and SD with SAF in the total population. Spearman rank correlation was used for the non-normally distributed data. Interaction analysis was used to investigate whether glucose metabolism status modifies the association of CV and SD with SAF. We conducted multiple linear regression analyses to examine the association of glucose variability with SAF in the total population and stratified by glucose metabolism status. Results are presented as crude analysis (Model 1). The fully adjusted model was adjusted for sex, age, BMI, HbA_1c_, serum creatinine, hypertension, total cholesterol:HDL-cholesterol ratio, smoking, alcohol intake, nutritional factors (energy intake and adherence to DHD summary score) and educational level. Interaction analysis was performed to test the effect of TATR in the association of CV and SD with SAF. Moreover, in order to check for the robustness of our results, we performed a sensitivity analysis by replacing CV and SD in our analysis with MAGE and MODD. In addition, a complete case analysis was performed.

Statistical analyses were performed using R version 4.2.1 using the following packages: tidyverse [21]; haven [22]; dplyr [23]; ggplot2 [24]; rcompanion [25]; ggpubr [24]; rstatix [26]; and table1 [27].

## Results

**Fig. 1:**
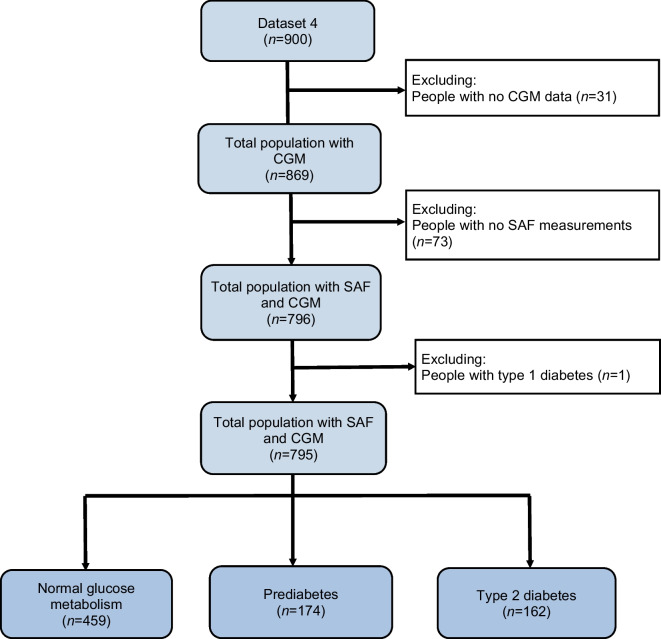
Flowchart of the study population

Figure 1 shows the flowchart of the study population including people with normal glucose metabolism (*n *= 459), people with prediabetes (*n *= 174) and people with type 2 diabetes (*n *= 162).

Table 1 shows the study population characteristics. In the overall population, there was a good balance between sexes (49% were female), the mean age was 59 ± 8.7  years, and the BMI was 27 kg/m^2^. Participants with type 2 diabetes were more often men, were older and had higher levels of BMI, HbA_1c_ and BP compared with the prediabetes and normal glucose metabolism groups. Finally, the DHD sum score was higher in people with normal glucose metabolism compared with those with prediabetes and type 2 diabetes, indicating that participants with normal glucose metabolism had a higher adherence to Dutch dietary guidelines.

**Table 1:** Descriptive characteristics of the total population and stratified by glucose metabolism status

Characteristic	Normal glucose metabolism (*N *= 459)	Prediabetes (*N *= 174)	Type 2 diabetes (*N *= 162)	*p* value
Male sex, *n* (%)	202 (44.0)	97 (55.7)	105 (64.8)	<0.001
Age, years	58.2 ± 8.9	61.1 ± 8.2	62.0 ± 7.8	<0.001
SAF value, AU	2.0 ± 0.4	2.1 ± 0.4	2.3 ± 0.6	<0.001
Diabetes duration, years	N/A	N/A	1 (0–3.0)	
HbA_1c_, mmol/mol	35.0 (33.0–38.0)	38.0 (35.0–40.0)	48.0 (42.0–54.0)	<0.001
HbA_1c_, %	5.3 (5.2–5.6)	5.6 (5.3–5.8)	6.5 (6.0–7.1)	<0.001
Serum total cholesterol, mmol/l	5.4 ± 1.0	5.4 ± 1.1	4.5 ± 1.1	<0.001
Total cholesterol:HDL-cholesterol ratio	3.3 (2.8–4.2)	3.8 (3.1–4.7)	3.6 (2.9–4.3)	<0.001
Systolic BP, mmHg	130 ± 17.6	137 ± 19.6	140 ± 15.4	<0.001
Diastolic BP, mmHg	73.6 ± 9.9	77.0 ± 10.3	78.1 ± 10.1	<0.001
Hypertension, *n* (%)	182 (39.7)	103 (59.2)	132 (81.5)	<0.001
GFR, ml/min per 1.73 m^2a^	81.1 ± 13.3	79.1 ± 13.0	80.2 ± 15.9	0.24
Insulin medication, *n* (%)	0 (0)	0 (0)	17 (10.5)	
Medication other than insulin, *n* (%)	0 (0)	0 (0)	84 (51.9)	
BP-lowering medication, *n* (%)	102 (22.2)	68 (39.1)	105 (64.8)	<0.001
BMI, kg/m^2^	25.6 ± 3.7	28.6 ± 4.3	29.7 ± 4.7	<0.001
Waist circumference, cm	91.7 ± 11.7	101 ± 11.9	105 ± 12.9	<0.001
Energy, kJ^b^	8702.7 (615)	8786.4 (660)	8535.4 (608)	0.7
Sum score Dutch Healthy Diet^b^	86.2 (16.5)	80.3 (15.3)	81.3 (15.2)	<0.001
Alcohol consumption, g/day^b^	7.9 (1.7–15.9)	9.7 (2.4–20.9)	6.0 (0.9–15.2)	0.01
Smoking status, *n* (%)^c^				0.12
Never	193 (42.0)	57 (32.8)	57 (35.2)	
Former	206 (44.9)	97 (55.7)	81 (50.0)	
Current	58 (12.6)	20 (11.5)	24 (14.8)	
Education category, *n* (%)^d^				0.02
Low	121 (26.4)	62 (35.6)	62 (38.3)	
Medium	129 (28.1)	45 (25.9)	47 (29.0)	
High	205 (44.7)	67 (38.5)	53 (32.7)	

Data are presented as means ± SD, median (IQR), or *n* (%)

^a^CKD-EPI equation estimated GFR using serum creatinine only

^b^Number of missing data: *n *= 147

^c^Number of missing data: *n *= 2

^d^Number of missing data: *n *= 4

Table 2 presents the glucometrics data of the total population and stratified by glucose metabolism status. The TIR for the total population was 99.8% and there were no measurements below 3.9 mmol/l or above 14 mmol/l. As expected, participants with type 2 diabetes had lower TIR and the highest values of SD and CV compared with the other two groups.

**Table 2:** Glucometrics data in the total population and stratified by glucose metabolism status

Glucometrics	Normal glucose metabolism (*N *= 459)	Prediabetes (*N *= 174)	Type 2 diabetes (*N *= 162)	*p* value
TIR (%)	100 (99.5–100)	99.8 (98.6–100)	91.8 (79.5–98.8)	<0.001
TATR (%)	1.4 (0.1–4.0)	5.0 (2.1–12.1)	34.1 (16.2–63.0)	<0.001
TAR (%)	0 (0–0)	0 (0–0.5)	7.4 (0.9–20.0)	<0.001
SD (mmol/l)	0.7 (0.6–0.9)	0.9 (0.7–1.1)	1.5 (1.1–2.0)	<0.001
CV (%)	12.6 (10.7–15.0)	14.7 (12.2–17.2)	19.3 (16.0–24.0)	<0.001
MAGE (mmol/l)	1.4 (1.2–1.9)	2.0 (1.6–2.5)	3.4 (2.4–4.6)	<0.001
MODD (mmol/l)	0.7 (0.6–0.8)	0.8 (0.7–1.0)	1.4 (1.1–1.8)	<0.001

Data are presented as median (IQR)

The mean SAF values were statistically significant between the glucose metabolism groups. The type 2 diabetes population had higher SAF values compared with the other two groups and participants with prediabetes had higher SAF values (mean ± SD 2.3 ± 0.6 vs 2.1 ± 0.4 AU, *p *= 0.014) than those with normal glucose metabolism (vs 2.0 ± 0.4 AU, *p *= 0.007).

**Fig. 2:**
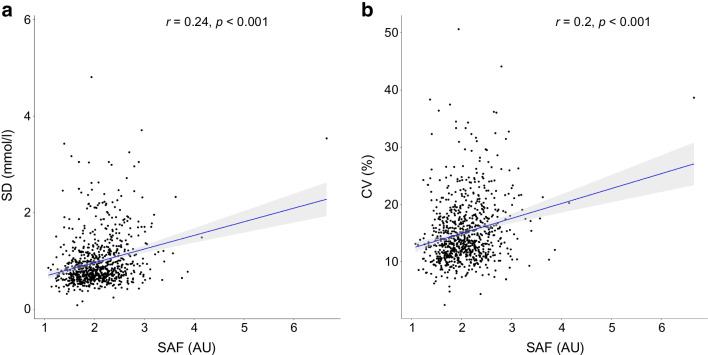
Correlations between SAF, sensor SD and sensor CV in the total population

In the total study population, glucose variability was significantly associated with SAF in the crude model (Fig. 2a, b), with a β (95% CI) of 0.018 (0.012, 0.024) for CV and 0.223 (0.163, 0.283) for SD (Table 3). In the fully adjusted model, we observed that for every 1% increase of CV and 1 mmol/l of SD, SAF increased by 0.014 AU and 0.152 AU, respectively (*p*<0.001 for both). Adjustments for potential confounders such as age, sex, BMI and cardiovascular risk factors, nutritional factors and educational level did not change the associations of CV and SD with SAF (Table 3). We observed a significant correlation between TATR and SAF in the total study population (electronic supplementary material [ESM] Fig. 1a). However, according to the interaction analysis, TATR did not affect the association of CV and SD with SAF (ESM Table 1).

**Table 3:** Multiple linear regression to test the association of sensor CV and SD with SAF in the total population and stratified by glucose metabolism status

Glucometrics	Crude model^a^			Fully adjusted model^b^			*p*_interaction_	*p*_interaction_
	*N*	β	CI	*N*	β	CI	Prediabetes vs NGM	T2DM vs NGM
CV (%)								
Total population	795	0.018	0.012, 0.024*	644^c^^,d,e^	0.014	0.005, 0.017*	0.73	0.28
NGM	459	0.011	0.002, 0.021*	344^c^^,d,e^	0.009	0.000, 0.019*	-	-
Prediabetes	174	0.011	−0.004, 0.025	155^c^	0.006	−0.010, 0.023	-	-
T2DM	162	0.013	−0.000, 0.026	145^c^	0.012	−0.001, 0.026	-	-
SD (mmol/l)								
Total population	795	0.223	0.163, 0.283*	644^c^^,d,e^	0.152	0.088, 0.217*	0.56	0.68
NGM	459	0.249	0.099, 0.400*	344^c^^,d,e^	0.165	0.012, 0.318*	-	-
Prediabetes	174	0.177	−0.005, 0.360	155^c^	0.087	−0.113, 0.289	-	-
T2DM	162	0.142	0.013, 0.271*	145^c^	0.177	0.044, 0.309*	-	-

^a^Crude model: Model 1

^b^Fully adjusted model: Model 1 + age, sex, CVD risk factors, nutritional factors and educational level (CVD risk factors: GFR, BMI, smoking status, HDL - cholesterol ratio, BP-lowering medication; nutritional factors: DHD sum minus alcohol, alcohol consumption; education level; and dietary factors: energy)

^c^Number of missing data: *n *= 147 for DHD minus alcohol, alcohol consumption and energy

^d^Number of missing data: *n *= 2 for smoking status

^e^Number of missing data: *n *= 4 for educational level

^*^p<0.05

NGM, normal glucose metabolism; T2DM, type 2 diabetes

Interaction analysis showed no effect modification of glucose metabolism status in the association of CV and SD with SAF. When stratified by glucose metabolism group and after full adjustments, CV was significantly associated with SAF in the normal glucose metabolism group, and SD was significantly associated with SAF in both the normal glucose metabolism group and the type 2 diabetes group. We found no such associations in the prediabetes group (Table 3).

In sensitivity analysis, we replaced SD and CV by MAGE and MODD, which also represent glucose variability, but these did not materially change the results (ESM Figs 2, 3). Finally, the complete case analysis did not change the results, except that for the type 2 diabetes group in the crude model the results were statistically significant (ESM Table 2).

## Discussion

In this study, we found that measurement of AGEs in the skin by SAF was positively associated with glucose variability, as derived from CGM in a large, extensively phenotyped, population. SAF was highest in participants with type 2 diabetes and still higher in people with prediabetes compared with those with normal glucose metabolism. However, the association between measures of glucose variability and SAF was largely independent of the glucose metabolism status and did not materially change after adjusting for a range of confounders. The association between glucose variability and SAF was also not driven by (slightly) elevated glucose values.

We found that both glucose variability and SAF were highest in the participants with type 2 diabetes and lowest in those with normal glucose metabolism, while those with prediabetes had levels of these outcomes that were between these two groups. These results for SAF are in accordance with previous data on SAF in people with type 2 diabetes [28] and those with prediabetes [18, 28–31]. Although, the size of the association between measures of glucose variability and SAF was small in our study, it should be acknowledged that this was observed in a population with normal to slightly elevated glucose variability, even in those with type 2 diabetes. The relatively short duration of diabetes in the affected population, with a total of 66 people (41%) being diagnosed on the basis of the OGTT performed at study entry, may have played a role here. Additionally, glucose-lowering medication or lifestyle changes used by people with type 2 diabetes to control for glucose fluctuations may have interacted with the associations, since adherence to a healthy diet may reduce SAF [32].

Although the interaction analysis showed that the association between glucose variability and SAF did not differ between the three subgroups, in the stratified analysis we found the association with SD to be apparent in participants with type 2 diabetes and those with normal glucose metabolism, but not in those with prediabetes. These somewhat discrepant findings may be explained by the higher glucose variability in the type 2 diabetes group and the much larger sample size of the normal glucose metabolism group, with the stratified analysis being underpowered to show effects in the prediabetes subgroup. We may speculate upon whether extrapolating our findings to diabetes populations with higher glucose variability may have resulted in SAF measurements that are more clinically relevant but this needs to be confirmed. Adjustments for sex in the model did not materially change the findings, meaning that these findings apply to both sexes without an indication that these differ between men and women.

We found no evidence of effect modification of mild hyperglycaemia, as defined by glucose values above 7.8 mmol/l, between the association of either CV or SD with SAF. We used this relatively low cut-off value rather than TAR (i.e. glucose >10 mmol/l), due to the low number of participants reaching such elevated glucose levels. It has been shown previously that there are associations between SAF and HbA_1c_ in people with type 1 diabetes [33] and in those with type 2 diabetes [34]. Our data extend these findings by showing that varying levels of only mildly elevated glucose levels, in the absence of increased HbA_1c_ as a reflection of chronic hyperglycaemia, is associated with increased levels of SAF.

Glucose variability is of high interest because it is associated with increased cardiovascular complications in people with diabetes [35] and possibly also in those with prediabetes [7]. Our findings regarding the association between glucose variability, as measured by CGM, and SAF, suggest involvement of AGEs as a potential underlying mechanism explaining how glucose variability increases the risk of CVD. However, the range of CV in our study population was well below the currently recommended threshold of CV, set at 36% for people with diabetes [36]. Although this threshold has been reported to distinguish between ‘stable’ and ‘unstable’ blood glucose levels, it should be acknowledged that this distinction is based on its association with hypoglycaemia frequency [37], rather than with the risk of CVD. Previously, we used the same cohort to report an association between glucose variability and aortic stiffness [38], also a determinant of CVD. When these data can be extended to actual CVD events, they would provide arguments to aim for lower CV, at least in people with (type 2) diabetes, than currently recommended.

Both CGM and SAF are measured in the same compartment (i.e. the subcutaneous area), indicating that the formation of AGEs occurs locally rather than originating in the blood compartment. Local formation of AGEs could explain previous observations regarding the presence of AGEs in the endothelium as well as in cardiac tissue, which may play a role in the development of heart failure [39, 40]. However, although SAF is used as a reflection of AGEs in the skin, we cannot exclude the possibility that the association of glucose variability with SAF is due to other fluorophores such as NADH [18].

A strength of this study is the extensive phenotyping of the participants of the Maastricht Study, which allowed us to adjust for multiple confounders. Moreover, we had CGM and SAF data from healthy individuals and individuals with prediabetes and type 2 diabetes, which gave us the opportunity to compare the three groups. There are also limitations. As discussed above, the sample size was relatively small and may have been too small to observe statistically significant associations for the diabetes subgroup. Second, there might be a selection bias towards people with healthier habits and therefore less glucose variability, giving their willingness for participation in the Maastricht Study, which raises questions with respect to generalisability. Indeed, the type 2 diabetes population was well controlled, and their glucose variability was low. Future studies should be repeated in a more diverse type 2 diabetes population with greater glucose variability. Third, CGM data were collected over a period of 1 week, whereas the consensus document recommends gathering data over a minimum of 2 weeks to better reflect longer-term glucose profiles, including glucose variability, at least in people with diabetes [36, 41]. Finally, the cross-sectional nature of our study does not allow for making assumptions about causality.

In conclusion, we found that glucose variability, as measured in interstitial fluid, is positively associated with SAF. This association is independent of sociodemographic factors, CVD risk factors and nutritional factors and of slightly elevated glucose levels and does not differ according to the presence or absence of type 2 diabetes. These data suggest that the formation of AGEs contributes as a mechanism underlying the harmful cardiovascular effects of glucose variability. Future studies are needed to investigate how glucose variability may induce formation of AGEs from a mechanistic point of view and whether this may help to explain how glucose variability increases the risk of harmful cardiovascular effects.

## Supplementary Information

Below is the link to the electronic supplementary material.

ESM (PDF 218 KB)

## Data Availability

The data of this study derive from the Maastricht Study but restrictions apply to the availability of these data, which were used under license for the current study. Data are, however, available from the corresponding author upon reasonable request and with permission of the Maastricht Study management team.

## Abbreviations

AU: Arbitrary units
CGM: Continuous glucose monitoring
DHD: Dutch Healthy Diet
FFQ: Food frequency questionnaire
MAGE: Mean amplitude glycaemic excursion
MODD: Mean of daily differences
SAF: Skin autofluorescence
TAR: Time above range
TATR: Time above tight range
TIR: Time in range
