# Supplementary material for: Glucose variability measured by continuous glucose monitoring is associated with skin autofluorescence: the Maastricht Study
Source: Diabetologia. 2025 Jun 20;68(9):1937–46. doi: 10.1007/s00125-025-06469-5 (PMC12361322; doi:10.1007/s00125-025-06469-5)
Supplement: Supplementary file 1 — ESM (PDF 218 KB) [file 125_2025_6469_MOESM1_ESM.pdf]

Electronic supplementary material (ESM)

ESM Table 1

Multiple linear regression to test the association of sensor CV and SD with SAF in the total population

		**Crude model[a]**			**Fully adjusted model[b]**			**P interaction TATR vs no TATR**
	N	*β*	*CI*	*N*	*β*	*CI*	*N*	
**Coefficient of Variation (%)**								
Total population	795	0.018	0.012, 0.024	644[c,d,e]	0.014	0.005, 0.017	628	0.56
**Standard deviation (mmol/l)**								
Total population	795	0.223	0.163, 0.283	644[c,d,e]	0.152	0.088, 0.217	628	0.77

[a]Crude model: Model 1

[b]Fully adjusted model: Model 1 + age, sex, CVD risk factors, nutritional factors and educational level (CVD risk factors: GFR, BMI, smoking status, HDL - cholersterol ratio, blood pressure lowering medication; nutritional factors: DHD sum minus alcohol, alcohol consumption; education level; and dietary factors: energy).

[c]Number of missings (DHD minus alcohol, alcohol consumption, and energy): n=147; [d]Number of missings (smoking status): n=2; [e]Number of missings (educational level): n=4

*p<0.05.

ESM Table 2

Multiple linear regression to test the association of sensor CV and SD with SAF in the total population and stratified by glucose metabolism status with complete case analysis.

	**Crude model[a]**				**Fully adjusted model[b]**				**P $_{interaction}$ Prediabetes vs NGM**	**P $_{interaction}$ T2DM vs NGM**
	N	*β*	*CI*	*P*	*N*	*β*	*CI*	*P*		
**Coefficient of Variation (%)**										
Total population	644	0.019	0.013, 0.025	0.00	644	0.014	0.005, 0.017	0.00	0.73	0.28
NGM	344	0.016	0.005, 0.026	0.00	344	0.009	0.000, 0.019	0.05	**-**	**-**
Prediabetes	155	0.009	-0.006, 0.025	0.25	155	0.006	-0.010, 0.023	0.43	-	-
T2DM	145	0.016	0.002, 0.029	0.02	145	0.012	-0.001, 0.026	0.07	-	-
**Standard Deviation (mmol/l)**										
Total population	644	0.235	0.171, 0.299	0.00	644	0.152	0.088, 0.217	0.00	0.56	0.68
NGM	344	0.324	0.156, 0.491	0.00	344	0.165	0.012, 0.318	0.03	-	-
Prediabetes	155	0.156	-0.036, 0.348	0.11	155	0.087	-0.113, 0.289	0.39	-	-
T2DM	145	0.193	0.059, 0.326	0.00	145	0.177	0.044, 0.309	0.00	**-**	**-**

[a]Crude model: Model 1

[b]Fully adjusted model: Model 1 + age, sex, CVD risk factors, nutritional factors and educational level (CVD risk factors: GFR, BMI, smoking status, HDL - cholersterol ratio, blood pressure lowering medication; nutritional factors: DHD sum minus alcohol, alcohol consumption; education level; and dietary factors: energy).

NGM, normal glucose metabolism; T2DM, type 2 diabetes

ESM Fig. 1

Correlation between SAF and TATR in the total population and stratified by glucose metabolism status

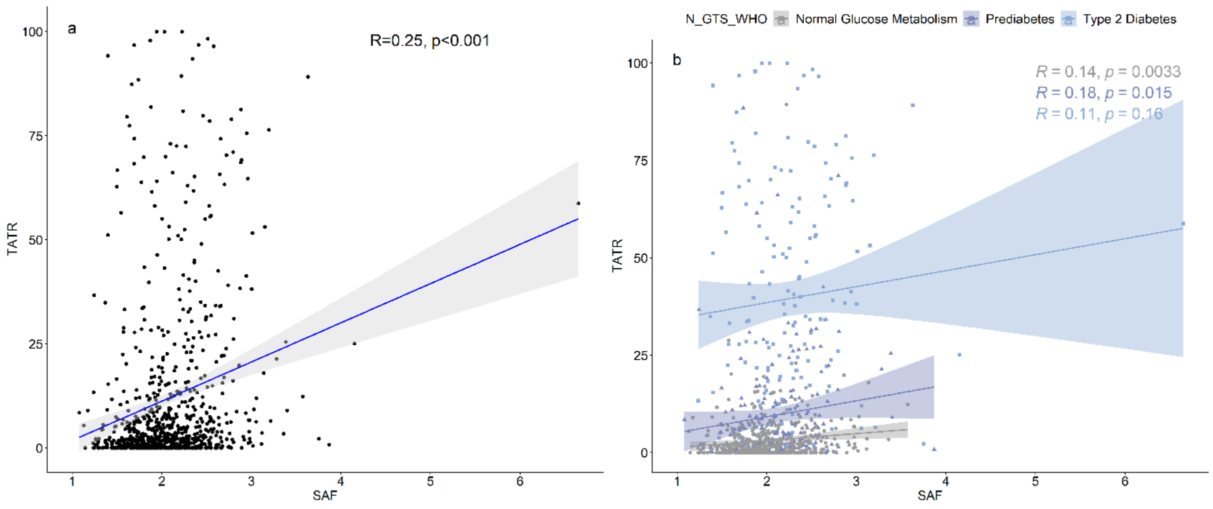

SAF, skin autofluorescence; TATR, time above 7.8mmol/l; N_GTS_WHO, glucose tolerance status

ESM Fig. 2

Correlation between MAGE and SAF in the total population and stratified by glucose metabolism status

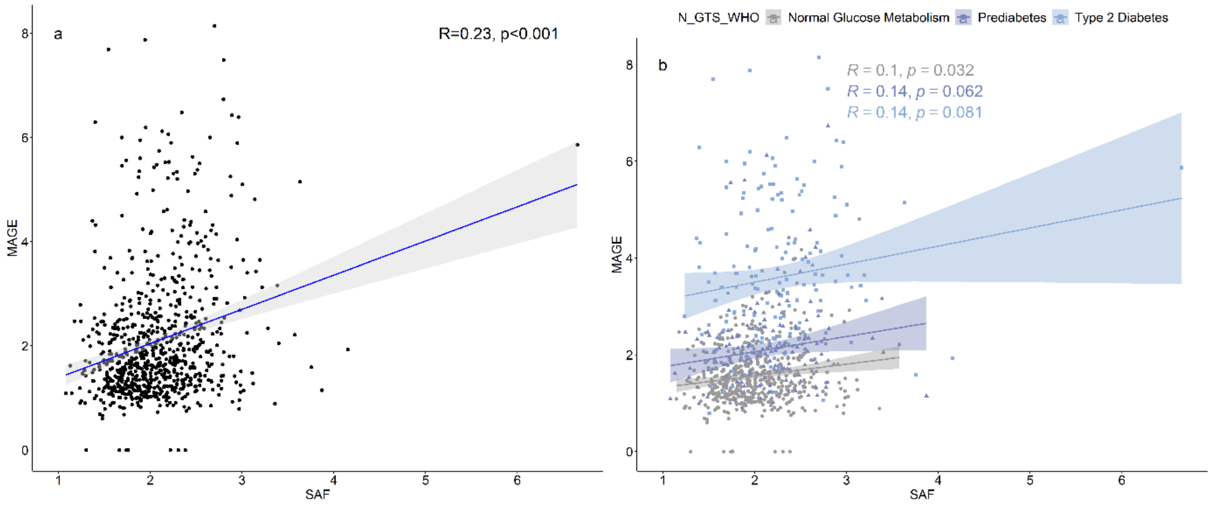

MAGE, mean amplitude of glycaemic excursion; SAF, skin autofluorescence; N_GTS_WHO, glucose tolerance status

ESM Fig. 3

Correlation between MODD and SAF in the total population and stratified by glucose metabolism status

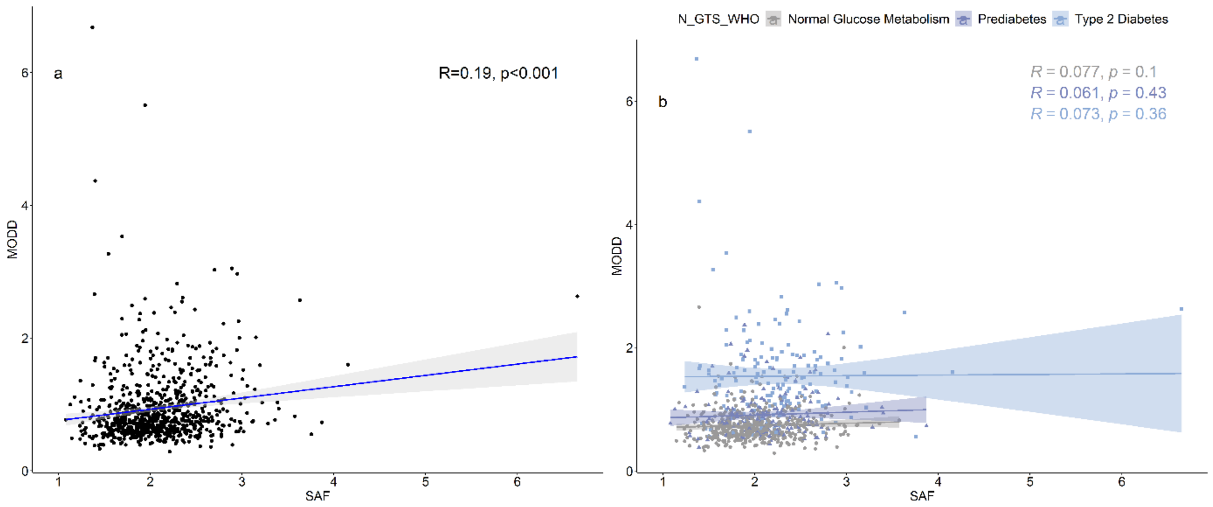

MODD, mean of daily differences; SAF, skin autofluorescence; N_GTS_WHO, glucose tolerance status
